# Atrial fibrillation and cancer: Two peas in a pod

**DOI:** 10.1016/j.rpth.2023.100073

**Published:** 2023-02-16

**Authors:** Orly Leiva

**Affiliations:** Division of Cardiology, Department of Medicine, New York University Grossman School of Medicine, New York City, New York, USA

To the Editor,

The recently published study by Ay et al. [1] titled “Atrial fibrillation and cancer: prevalence and relative risk from a nationwide study” was read with great interest. The authors should be applauded for their efforts in further elucidating the risk of atrial fibrillation (AF) in patients with cancer and vice versa. Indeed, there appear to be several common pathophysiologic similarities between AF and cancer, including inflammation as well as off-target and adverse effects of cancer therapies [2]. Although the study by Ay et al. [1] is an important addition to the literature on the epidemiology of AF in patients with cancer, there remain several unanswered questions.

The authors described the highest risk of AF in patients with hematologic malignancies with a relative risk of 9.15. However, hematologic malignancies are a diverse group of disorders with widely ranging presentations and treatments, and the current study did not discern the risk of AF by the type of hematologic malignancy. This is particularly important given that patients with chronic lymphocytic leukemia are often treated with Bruton kinase inhibitors, including ibrutinib, which carry significant risks of AF [3]. The identification of other hematologic malignancies at a high risk of AF is clinically important because traditional risk scores, such as the CHADS2VASC and HAS-BLED scores, may not perform as well in these patient populations, and further investigation is warranted [4]. Additionally, in other studies, lung cancer has been associated with the highest risk of AF, perhaps because of its proximity to the heart and the effects of intrathoracic surgery and radiation therapy on the heart [5]. Lack of granularity in specific cancer types and therapies (previous surgery, chemotherapy used, etc) is a limitation of this study and most studies involving large, nationwide databases. However, it is a fertile ground for further investigation in order to have a more complete understanding of the risk of AF in patients with cancer and the development of more accurate risk scores and prediction models. Large, prospective registry studies are needed that include granular data on cancer types, genetics, and treatments in order to have a more nuanced understanding of the risk of AF in this patient population.
